# Triage of Acute Ischemic Stroke in Confirmed COVID-19: Large Vessel Occlusion Associated With Coronavirus Infection

**DOI:** 10.3389/fneur.2020.00353

**Published:** 2020-04-21

**Authors:** Pouria Moshayedi, Timothy E. Ryan, Lucido Luciano Ponce Mejia, May Nour, David S. Liebeskind

**Affiliations:** ^1^Department of Neurology, University of California, Los Angeles, Los Angeles, CA, United States; ^2^Department of Interventional Neuroradiology, University of California, Los Angeles, Los Angeles, CA, United States

**Keywords:** COVID-19, stroke—diagnosis, therapy, large vessel occlusion (LVO), disease transmission, triage

## Abstract

The outbreak of COVID-19 has posed a significant challenge to global healthcare. Acute stroke care requires rapid bedside attendance, imaging, and intervention. However, for acute stroke patients who have a diagnosis of or are under investigation for COVID-19, the concern for nosocomial transmission moderates operational procedures for acute stroke care. We present our experience with an in-hospital stroke code called on a COVID-19-positive patient with a left middle cerebral artery syndrome and the challenges faced for timely examination, imaging, and decision to intervene. The outlook for the ongoing COVID-19 pandemic necessitates the development of protocols to sustain timely and effective acute stroke care while mitigating healthcare-associated transmission.

## Introduction

Rapid attendance at the patient bedside, clinical exam, and timely imaging studies have been emphasized in the care of acute ischemic stroke patients, but the global pandemic outbreak of COVID-19 (1) has created novel and significant challenges to acute stroke care. Protocols to sustain acute stroke care for COVID-19 patients while mitigating nosocomial transmission are needed. In this report, we share unique challenges in treating a COVID-19-positive patient with acute ischemic stroke due to occlusion of the left middle cerebral artery. We also discuss the current evidence and recommendations to decrease healthcare-associated transmission in acute clinical examination, imaging, and interventional procedures in acute stroke patients with a diagnosis of COVID-19.

## Case Presentation

A patient in his 8th decade of life was admitted to our facility with acute chest pain, diaphoresis, and hypotension with ST-elevation myocardial infarction (STEMI). He underwent coronary angioplasty followed by stent deployment and was admitted to the coronary care unit (CCU). Transthoracic echocardiogram revealed left ventricle (LV) ejection fraction of 10–15% and LV thrombosis measuring 2.8 cm × 1.1 cm, prompting initiation of IV heparin. He also had bilateral lower limb paresthesia and loss of temperature and arterial pulses, raising concern of ischemic limbs, with lower extremity arterial Doppler confirming occlusion in multiple arterial segments.

Due to his presentation with shortness of breath, he was designated as a Patient Under Investigation (PUI) for COVID-19, and, the day following admission, his PCR testing of respiratory secretions showed evidence of 2019-nCOV RNA. He was intubated due to hypoxemic respiratory failure. Standard facility protocols for droplet isolation in a negative pressure room were implemented. The patient was extubated on day 4.

On day 5, an in-hospital stroke code was called after the bedside nurse found him to have an inability to speak, right-sided weakness, and right-sided facial droop, with last known well time the night before. The stroke team arrived, and one team member wore personal protection equipment including an N95 respirator mask, goggle, gown, and gloves and entered the patient's room to perform an examination, which revealed an inability to speak or comprehend, a conjugate gaze preference toward the left side, no blink to threat on the right side, and no movement in the right arm and leg. Since the patient presented with a large vessel occlusion stroke syndrome within an extended time window, advanced multimodal imaging was indicated to measure infarct volume as well as salvageable tissue. According to the institutional policy for acute stroke codes, as well as the patient's renal failure prohibiting the use of iodine contrast for CT perfusion studies, he was transported to MRI. The institutional protocol for transferring patients in aerosol isolation was applied by having the patient wear a surgical face mask. Laboratory results were notable for renal failure, normal platelet count, and activated partial thromboplastin time (PTT) >85.5 while on heparin.

Brain MRI, obtained without contrast administration (due to severe renal failure) and reviewed during image acquisition, revealed a 60-cc acute infarct in the left insular, temporal, parietal, and frontal lobes, as well as smaller acute infarcts in the right caudate and left cerebellar hemisphere. MRI also showed evidence of hemorrhagic conversion in the left fronto-temporal territory. MR angiogram showed occlusion of the left middle cerebral artery proximal M1 segment.

The patient was not a candidate for thrombolysis as he had elevated activated PTT on heparin, in addition to an unknown time of stroke onset and evidence of large and established infarction with a small region of hemorrhagic transformation. Endovascular thrombectomy was considered but not pursued given the large infarct size and evidence of hemorrhagic transformation since reperfusion could have led to a much larger intracranial hemorrhage with no meaningful clinical benefit. Following this ischemic stroke, and given other comorbidities including heart failure, cardiovascular shock, and ischemic bilateral lower limbs, discussions on goals of care were held with the family, and his care was focused on comfort according to the patient's wishes.

Following the completion of his MRI, the MRI suite was out of commission for about 3 h to complete disinfection.

## Discussion

Early reports from the COVID-19 pandemic have noted a 41% nosocomial infection rate (2), which highlights the importance of developing protocols for transfer, imaging, intubation, and surgical or endovascular procedures on COVID-19 patients presenting with acute stroke. The novelty of the COVID-19 pandemic outbreak means that there are few evidence-based and informed protocols. The current recommendations have centered on the airborne and direct contact methods of transmission (3).

To perform MRI or other imaging modalities, it is recommended to switch a patient's bed and accessories to MRI-compatible equipment in the patient room with enhanced airborne precautions rather than in the MRI suite. Designation of a single entry point to imaging facilities and limiting other traffic through the healthcare facility is preferred (4). Installment of physical barriers and the donning of N95 masks, face protection, and gloves for physicians or ancillary staff en-route are recommended.

Discussions on safe pre-operative procedures are underway (5). Studies published before the COVID-19 outbreak noted that a high-flow nasal cannula, non-invasive ventilation by masks with optimized vent holes, or a helmet connected to a double-limb circuit may lower airborne transmission (6), but closed-circuit ventilation with use of filters is regarded as a safer option. Given the relatively low risk of operative site infection, endovascular suites capable of conversion to negative pressure facilities appear to be ideal solutions.

The COVID-19 pandemic outbreak has exhausted and overburdened healthcare systems. In a situation of extreme facility shortages, triaging COVID-19-positive patients with acute stroke based on severity and extent of their pre-morbidities is a grim but unavoidable necessity. In acute stroke patients presenting with symptoms of large vessel occlusion in an extended time window, and therefore requiring advanced imaging, CT perfusion has been shown to be equally capable of selecting patients for endovascular thrombectomy when compared with MRI (7). The time required for disinfection protocols to be conducted may require judicious use of MRI for acute stroke patients. In the face of the current pandemic, our institutional policy for acute stroke care has changed and now prioritizes CT perfusion over MRI.

## Data Availability Statement

All datasets presented in this study are included in the article/supplementary material.

## Ethics Statement

The studies involving human participants were reviewed and approved by UCLA IRB. Written informed consent for participation was not required for this study in accordance with the national legislation and the institutional requirements.

## Author Contributions

PM and DL: conception and design and administrative/technical/material support. PM: acquisition of data and drafting the article. All authors: analysis and interpretation of data, critically revising the article, and reviewed submitted version of manuscript. DL: approved the final version of the manuscript on behalf of all authors and study supervision.

## Conflict of Interest

DL is consultant to Cerenovus, Genentech, Stryker and Medtronic as Imaging Core Lab. The remaining authors declare that the research was conducted in the absence of any commercial or financial relationships that could be construed as a potential conflict of interest.
